# Combination of trained immunity and specific inactivated vaccine against *S*. Choleraesuis infection in pigs

**DOI:** 10.3389/fvets.2026.1879508

**Published:** 2026-07-23

**Authors:** Lidia Sánchez-Morales, Marta Pérez-Sancho, Teresa García-Seco, Ana Balseiro, Andrea Pérez-Domingo, Aránzazu Buendía, Alberto Díez-Guerrier, Álvaro Fernández-Manzano, David Relimpio, María Ugarte-Ruiz, María de los Ángeles Risalde, Christian Gortázar, Lucas Domínguez

**Affiliations:** 1 VISAVET Health Surveillance Centre, Complutense University of Madrid, Madrid, Spain; 2 Department of Animal Health, Faculty of Veterinary Medicine, Complutense University of Madrid, Madrid, Spain; 3 Department of Animal Health, Faculty of Veterinary Medicine, University of León, León, Spain; 4 Instituto de Ganadería de Montaña (IGM, CSIC-ULE), León, Spain; 5 MAEVA SERVET S.L., Alameda del Valle, Spain; 6 SaBio Instituto de Investigación en Recursos Cinegéticos IREC (CSIC-UCLM-JCCM), Ciudad Real, Spain; 7 Departamento de Anatomía y Anatomía Patológica Comparadas y Toxicología, Grupo de Investigación en Sanidad Animal y Zoonosis (GISAZ), UIC Zoonosis y Enfermedades Emergentes ENZOEM, Universidad de Córdoba, Córdoba, Spain

**Keywords:** cross-protection, mycobacteria, *S*. Choleraesuis, *Salmonella*, trained immunity, vaccine

## Abstract

**Introduction:**

*Salmonella enterica* subsp. *enterica serovar* Choleraesuis is a pathogen that can cause severe systemic disease and remains a challenge in swine production due to its impact on animal health, economic losses, and zoonotic risk. While vaccination is a key control tool, current vaccines often provide limited protection to the targeted (and untargeted) serovar. Trained immunity has gained attention as a possible way to enhance non-specific protection.

**Methods:**

This study explored whether a porcine-derived BCG strain (dpB), administered live or inactivated, could improve the protective effect of a conventional *S*. Choleraesuis vaccine in pigs, not only against homologous challenge but also against heterologous serovars.

**Results:**

Pigs immunized with inactivated dpB in conjunction with the *S*. Choleraesuis vaccine showed a trend toward better bacterial control, with the lowest levels of *S*. Choleraesuis recovered from feces and ileocecal lymph nodes. Additionally, this group demonstrated a slight improved immune reactivity to heterologous serovars and a balanced cytokine response, without indications of excessive inflammation. In contrast, animals that received live dpB in addition to the vaccine showed the most severe pulmonary lesions, lowest phagocytic activity and a dysregulated cytokine profile.

**Discussion:**

These results suggest that inactivated dpB may modulate immune responses when administered together with a conventional *S*. Choleraesuis vaccine and could contribute to improved bacterial control. However, the observed effects were generally modest and require confirmation in larger studies. These preliminary results support further investigation of trained immunity-based approaches as potential strategies added to conventional vaccination against *Salmonella* in swine.

## 1 Introduction

*Salmonella enterica* remains a pathogen of concern in both veterinary and public health, particularly within the swine sector. Among its several serovars, *Salmonella enterica subsp. enterica* serovar Choleraesuis (*S*. Choleraesuis), is highly adapted to pigs and represents great importance because of its great virulence producing systemic disease marked by septicemia, pneumonia, and enterocolitis (1). The clinical signs observed in swine consist of fever, respiratory distress, diarrhea, and, in severe cases, sudden death, especially in the case of weaned piglets (2). The economic impact from Salmonella infections in pigs is substantial, due to lower weight gain, higher mortality, treatment costs, and trade restrictions in the industry (3). Additionally, the prevalence of *S*. Choleraesuis in the wild boar, its reservoir, is rising in Europe (4). In this line, outbreaks of antimicrobial resistant *S*. Choleraesuis have been reported in wild boar piglets in Spain (5).

In addition to its effect on animal health, *S*. Choleraesuis presents a zoonotic risk, especially regarding occupational contact or consumption of contaminated pork products (1, 6). Other serovars such as *S*. Typhimurium and its monophasic variant (1,4,[5],12:i:-) have also been isolated from pigs and are among the most common causes of Salmonella infection in humans in Europe and abroad (1, 7). Finally, the increasing prevalence of antimicrobial resistant Salmonella strains complicates control efforts (8, 9).

The existing control methods against Salmonella in swine production consist mainly in a combination of vaccination and biosecurity including hygiene practices. However, the effectiveness of current vaccines could be considered limited because they stimulate immune responses that are specific of serovar (1). Therefore, these vaccines typically fail to provide adequate protection against other Salmonella serovars (10, 11). On the other hand, live attenuated vaccines, such as those targeting *S*. Choleraesuis, have shown efficacy in mitigating clinical signs and bacterial shedding, but concerns regarding safety and limited heterologous protection remain present (12). In this line, inactivated vaccines tend to be safer, but may provide weaker immune responses, especially with no adjuvants or booster doses (11).

Given these limitations, there is a growing interest in alternative immunological strategies that can enhance innate immune responses and provide broader protection against swine Salmonella serotypes. One immunological approach is the induction of trained immunity, a form of innate immune memory characterized by epigenetic and metabolic reprogramming of innate immune cells such as monocytes, macrophages, and natural killer cells (13, 14). In contrast to adaptive immunity, trained immunity can increase responsiveness to subsequent infections, including those brought on by unrelated pathogens being not dependent on antigen specificity (15).

An established model for researching trained immunity is the Bacillus Calmette-Guérin (BCG) vaccine, originally developed against tuberculosis. In both human and animal models, BCG has demonstrated non-specific protection against a variety of pathogens, such as *Salmonella typhimurium*, *Listeria monocytogenes*, and *Candida albicans* (16, 17). However, studies investigating the role of trained immunity in controlling infectious diseases in veterinary species remain scarce. Despite this, recent research has highlighted its promising potential to enhance disease resistance in animals, offering a new direction for vaccine development beyond antigen-specific approaches (18). Thus, a recent study showed that piglets inoculated with heat-inactivated *Mycobacterium bovis* (HIMB) provided some protection against the *S*. Choleraesuis challenge (19). In addition to increased expression of proinflammatory cytokines like tumor necrosis factor alpha (TNF-α) and the mucosal chemokine CCL28, animals treated with HIMB showed improved weight gain, decreased respiratory symptoms, and attenuated pulmonary lesions (19). These findings suggested that mycobacterial preparations may modulate innate immune responses in pigs against Salmonella. The experiment did not explore, however, the potential synergistic effect of HIMB with conventional vaccines, or cross-protection against other significant Salmonella serovars.

In the present study, we aimed to expand these findings by exploring for the first time the immunostimulatory potential of a porcine-derived BCG strain (dpB) (20), administered in different forms (live and inactivated) combined with a *S*. Choleraesuis vaccine to amplify the efficacy to protect pigs against *Salmonella*. Specifically, we aimed to evaluate the efficacy of this combined immunization regimen in pigs against *S*. Choleraesuis challenge and induction of cross-reactive immune responses against other serovars with clinical relevance in swine such as *S*. Typhimurium and its monophasic variant (1,4,[5],12:i:-). By integrating the phenomenon of trained immunity into conventional vaccination, the major objective of this research was to provide novel insight into the development of broad-spectrum immunoprophylactic strategies for the control of salmonellosis in pigs.

## 2 Materials and methods

### 2.1 Bacterial strains, culture conditions, and preparation of inocula

#### 2.1.1 DpB preparation

##### 2.1.1.1 Live dpB

The dpB derivative, which shows a high homology with Danish CCUG 27863, obtained from swine and subsequent formulation, has showed relevant immunostimulatory properties (20). A vial of the frozen strain was inoculated into a liquid culture medium and incubated at 37 °C under aerobic conditions for 5 weeks. At the end of the culture period, the growth was collected with a pipette, centrifuged, washed with Phosphate Buffered Saline (PBS; Sigma-Aldrich, Misuri, USA), given a treatment of physical breakage with glass beads, and diluted with PBS until a homogeneous product was at approximately 1 McFarland [equivalent to approximately 106 colony forming units (CFU)/mL] according to commercial patrons employed (M-IDENT®-MacFARLAND, Laboratorios Microkit, Valdemorillo, Spain). A plate count was performed to determine the concentration of the prepared inoculum before and after inoculation of dpB in the swine (estimated concentrations 1–4 × 10^6^ CFU/mL). This concentration was selected based on previous studies using HIMB as an immunomodulatory agent, where similar bacterial loads (10^7^ CFU/mL) were employed with favorable results (19).

##### 2.1.1.2 Inactivated dpB

DpB was cultured for 5 weeks, then inactivated by heat, centrifuged, washed and resuspended in saline. The solution was sonicated and afterwards filtrated to remove larger particles. Thimerosal was added at 1% as a preservative due to its multidose presentation in a 50 mL vial.

#### 2.1.2 Clinical Salmonella strains

The *S*. Choleraesuis variant Kunzendorf was used as challenge strain, vaccine and antigen for seroaglutination and phagocytosis assays (19). The other strains used in this study were *S*. Typhimurium (4,5,12:i:1,2) and its monophasic variant 12:i:-.

All strains were initially growth in broth culture from pure cultures and incubated for 24 h (37 °C, 150 rpm in orbital agitation). The starter culture was transferred to a large-scale broth culture and incubated for 24 h (37 °C, 150 rpm in orbital agitation).

##### 2.1.2.1 Preparation of inactivated *S*. Choleraesuis vaccine

Inactivation was performed in two phases: (i) chemical inactivation: achieved by adjusting the product to 0.15% formaldehyde, using Formaldehyde 37–38% w/w stabilized with methanol (Panreac Química S.L.U., Castellar del Vallès, Barcelona, Spain), and (ii) heat inactivation: incubating for 30 min in a 75 °C water bath. The product was then refrigerated, and inactivation controls were conducted. Finally, the preparation was adjuvanted with 1% of 10% Thimerosal and 10% Montanide GEL 02 (Sigma-Aldrich, MO, USA), aliquoted and stored at 4 °C–6 °C until use. Final concentration obtained was 2.2 × 10^9^ CFU/mL.

##### 2.1.2.2 Preparation of *S*. Cholerasuis challenge inoculum

The *S*. Choleraesuis was diluted in PBS (Sigma-Aldrich, MO, USA) to achieve a final concentration of 2 × 10^8^ CFU/mL for the challenge. The preparation was then aliquoted and stored under refrigeration. Bacterial counts were confirmed both before (1.6 × 10^9^ CFU/mL) and after (2.8 × 10^9^ CFU/mL) the challenge to confirm the ensure product stability.

##### 2.1.2.3 Preparation of soluble antigens for cytokine stimulation

Soluble antigens were prepared from *S*. Choleraesuis, *S*. Typhimurium, and its monophasic variant using a standardized protocol. Cultures were heat inactivated for 30 min in a 75 °C water-bath, centrifuged and washed twice with sterile PBS. The final product was resuspended in PBS to a McFarland standard of 3–4 (equivalent to 2–4 × 10^9^ CFU/mL), ensuring a final stimulation dose of 10^8^ CFU/mL in blood by adding 100 μL of antigen per mL of blood.

##### 2.1.2.4 Preparation of formalin antigen for serological agglutination

Formalin-inactivated antigen from *S* . Choleraesuis was obtained by adding buffered formalin (1:1 ratio) to the culture and incubating at 37 °C with agitation (125 rpm) for 2 h. Following inactivation, cells were centrifuged to remove residual media and formalin, washed twice with PBS, and resuspended in PBS containing 1% Thimerosal. The suspension was adjusted to a final turbidity of 1.4 McFarland and stored at 4 °C in light-protected containers.

### 2.2 Experimental design

Animal care and procedures were performed by following the guidelines of good experimental practices according to Directive 2010/63/EU of the European Parliament and of the Council of 22 September 2010 on the protection of animals used for scientific purposes [amended by the Regulation (EU) 2019/1010 and Spanish laws (RD 53/2013)]. The protocol was approved by all the ethics committees required by legislation (reference PROEX101.8/24 of the Community of Madrid).

A total of 32 hybrid white swine from a Spanish agro-industrial with A4 health status (officially free from Aujeszky’s disease virus in accordance with Royal Decree 360/2009 of Spanish legislation) were received at the VISAVET facilities at approximately 27–30 days of age, corresponding to the post-weaning period. They were housed in four pens, one for each experimental group (8 animals/pen). Each pen measured 8 m^2^ and was equipped with straw, heat lamps, and basketballs to ensure animal wellbeing. Water and commercial feed from the source farm were provided *ad libitum*. The first day of procedures (immunization; 7 days after reception to allow properly housing accommodation of the animals) was also used to mark the animals with a numbered ear tag (Azasa, Madrid, Spain) for individual identification.

Figure 1 represents the four groups involved in the experiment, being: Group 1 (two doses of *S*. Choleraesuis vaccine), Group 2 (two doses of live dpB and *S*. Choleraesuis vaccine), Group 3 (two doses of inactivated dpB—*S*. Choleraesuis vaccine) and Group 4 (infection control without immunization).

**Figure 1:**
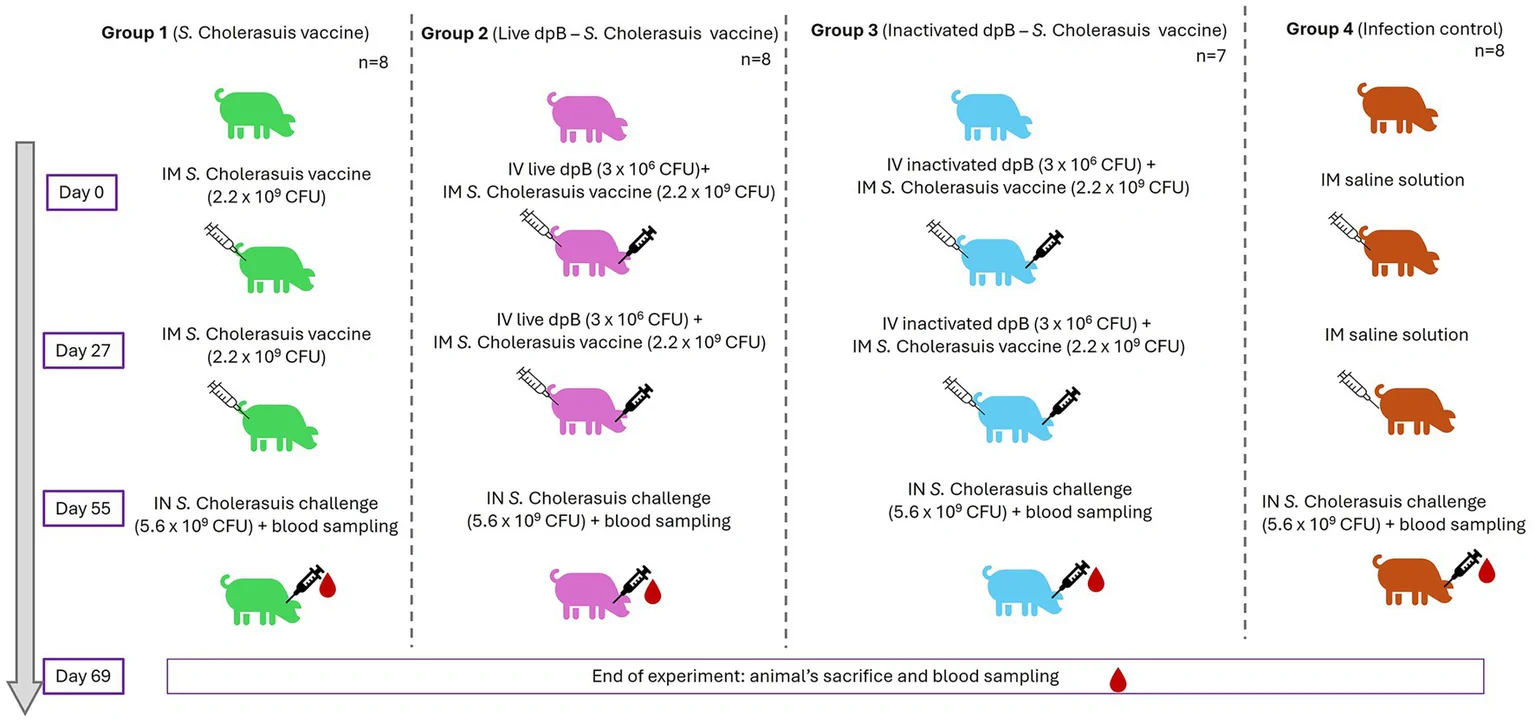
Experimental design and timeline, depicting the immunization-to-sacrifice period for the different groups of animals included in the study (IM, intramuscularly; IV: intravenously). Created with PowerPoint.

Before experimental procedures in days 0, 27 and 55, pigs were anesthetized with a combination of Zoletil 100 (tiletamine 50 mg/mL, zolacepam 50 mg/mL; Virvac [0.03 mL/kg]) and Medetor (medetomidine 0.85 mg/mL) [0.05 mL/kg] administered intramuscularly (IM) in the right gluteal region (unless animals which only received an intramuscular injection) according to previous protocol (21).

On day 0, animals from Groups 1, 2 and 3, received 1 mL of *S*. Choleraesuis vaccine (2.2 × 10^9^ CFU) via IM in the left gluteal region. Subsequently, 1 mL (3 × 10^6^ CFU) of either live or inactivated dpB was administered intravenously (IV) to animals from group 2 (dpB-*S*. Choleraesuis vaccine) and 3 (inactivated dpB- *S*. Choleraesuis vaccine) in the yugular vein. After 27 days, animals from groups 1, 2 and 3 received a booster of the same immunogens administered on day 0, following the same procedures as described previously. Group 4 remained as non-immunized control group.

After 55 days of the first immunization, 10 mL of blood from all animals from groups 1 to 4 was obtained from the jugular vein by using a vacutainer system and tubes with lithium heparin as anticoagulant. Thereafter, all animals from the four groups were challenged intranasally (IN) with 1 mL of 2.8 × 10^9^ CFU/mL of the *S*. Choleraesuis inoculum in each nostril by using an applicator for intranasal vaccine administration (total of 5.6 × 10^9^ CFU; Figure 1).

Following *S*. Choleraesuis challenge, animals were monitored daily in search of related clinical signs which were evaluated from 0 to 2 or 3 depending on severity (Table 1).

**Table 1:** Clinical score to evaluate clinical signs after *S*. Choleraesuis challenge.

Clinical score		
Respiratory signs	Dyspnea	0 absence
		1 moderate
		2 severe
	Sneezes	0 absence
		1 presence
	Coughs	0 absence
		1 presence
Depression		0 normal
		1 slightly depressed
		2 depressed
		3 moribund, does not stand up
Diarrhea		0 absence
		1 soft feces
		2 liquid feces

The endpoint criteria once the animals were infected were inability to stand for at least 10 s, severe despondency, lack of response to stimuli and/or lack of resistance to manipulation. One pig from group 3 died at day 7 due to non-related causes to the experiment, so results from this animal were not available. Fourteen days after the challenge and prior to the programmed slaughter of the animals, blood collection in heparin was carried out following the same protocol described earlier. After anesthesia described previously, animals were euthanized with T61 (MSD Animal Health, Spain; 200 mg embutramide, 50 mg mebezonium iodide, 5 mg tetracaine hydrochloride per mL; 0.3 mL/kg) IV in the jugular vein. Evaluation of gross lesions and sampling were carried out during the necropsy of the animals. For the histopathological study, lung and liver were introduced in 10% formaldehyde. Also, tonsils, lungs, ileocecal lymph node and intestinal content obtained from cecum (1 g/sample) were obtained for later culture.

### 2.3 Serological testing by microtiter agglutination

Titration of anti-*S*. Choleraesuis antibodies in serum samples obtained before challenge and before slaughter was performed by microtiter plate agglutination using formalin *S*. Choleraesuis antigen produced in-house.

For the assay, 50 μL of 1/10 initially diluted sera were ½ serially diluted in PBS-safranin in V-bottom 96-well microplates in a final volume of 50 μL. Same volume of antigen were added to each well, and plates were incubated at 37 °C overnight, followed by a 2-h refrigeration step before reading. Positive agglutination was defined as absence of antigen sedimentation. Serum titer was considered as the highest dilution at which positive agglutination was still observed.

### 2.4 Blood stimulation and cytokine measurements

A maximum of 6 h after sampling (to guarantee the viability of blood cells), three replicates of 1 mL of blood obtained from each animal in the *S*. Choleraesuis challenge day (before infection), were dispensed in 24-wells plate and stimulated with 100 μL of each antigen (*S*. Choleraesuis, *S*. Typhimurium monophasic variant, and *S*. Typhimurium) with a concentration of approximately 2.5–4 × 10^9^ CFU/mL. Thereafter, an incubation was carried out at 37 °C overnight in a humidity chamber. The next day, the plates were centrifuged (550–700 g for 10–15 min) and then the plasma was collected and frozen at −80 °C until processing.

Blood samples obtained at slaughter day were centrifuged for recovered plasma, without previous *S*. Choleraesuis *in vitro* stimulation, as in this point animals were previously challenged with *S*. Choleraesuis (“*in vivo* stimulated”).

Plasmatic cytokines as interleukin 1β (IL-1β), interleukin 6 (IL-6), TNF-α, interferon-gamma (IFN-γ), and anti-inflammatory as interleukin 10 (IL-10), were measured in plasma with ProcartaPlexTM kit by Thermo Fisher Scientific (MA, USA) and Bio-Plex 200 System (Bio-Rad Laboratories, Alcobendas, Madrid).

### 2.5 *S*. Choleraesuis culture for tissues

To assess *S*. Choleraesuis presence and distribution in tissues and feces, a semiquantitative culture method was performed using an internal protocol based on ISO 6579-2:2012 guidelines. Briefly, approximately 1–10 g of organ tissue or 10 g of cecum content were homogenized in buffered peptone water (BPW) at a 1:10 ratio and serial 10-fold dilutions were prepared to estimate bacterial load. After pre-enrichment at 37 °C for 18–24 h, a volume of the pre-enriched sample was inoculated onto modified semisolid Rappaport–Vassiliadis (MSRV) medium for selective enrichment. After incubation, samples showing comparable growth with Salmonella were cultured onto Brilliance™ Salmonella Agar for confirmation. CFU per gram of sample were estimated based on the highest dilution showing typical Salmonella growth (22).

### 2.6 Phagocytosis assay

Phagocytosis, represented as CFU/mL, indicates the capacity of neutrophils and monocytes in the animals’ blood to eliminate bacteria.

Phagocytosis assays were performed in preinfection (day 55) and final (day 69) points from immunized groups (1–3). Three 450 μL replicates of whole blood from each animal were recovered. Each replicated was inoculated with 50 μL from the different Salmonella strains: (*S*. Choleraesuis, *S*. Typhimurium monophasic variant, and *S*. Typhimurium). Additionally, a negative control was prepared by substituting the blood with PBS, and immediately (time 0) serial 10-fold dilutions were performed in PBS and plated in duplicate on blood agar (100 μL) using a Drigalski spreader and incubated at 37 °C (23, 24).

Subsequently, both blood samples and negative controls, were placed on a rotative shaker and incubated at 37 °C for 2 h. After incubation, serial 10-fold dilutions and plate cultures were performed. After 24 h of incubation at 37 °C, the colonies on the plates were counted (23, 24).

### 2.7 Histopathological study

Tissues were fixed in 10% neutral formalin for 48 h. The samples were automatically processed (Citadel 2000 Tissue Processor, Thermo Fisher Scientific, Waltham, MA, USA) and embedded in paraffin (HistoStar Embedding Workstation, Thermo Fisher Scientific, Waltham, MA, USA). Consecutive sections of 4 μm thickness were obtained using a microtome (FinesseMe+, Thermo Fisher Scientific, Waltham, MA, USA). Sections were stained with hematoxylin-eosin (HE). Finally, samples were mounted (CTM6 Coverslipper, Thermo Fisher Scientific) and evaluated for histopathological alterations under a Leica DM2000 microscope (Leica Microsystems, Wetzlar, 162 German). A single-blind study was conducted by the pathologist evaluating the following lesions in lungs and liver. In lungs, congestion, alveolar oedema, hemorrhage and, broncopneumonia were evaluated, assigning a value between 0 and 3 based on bronchitis, bronchiolitis and interstitial pneumonia severity, i.e., extension and grade of inflammatory infiltrate, mainly consisted of lymphocytes and macrophages; 0: no lesion; 1: mild lesions consisted of small areas of mild multifocal bronchopneumonia; 2: moderate lesions consisted of bronchopneumonia with a more widespread distribution; 3: severe lesions in which necrosis was also observed. In liver, hepatitis was evaluated, assigning a value between 0 and 2: 0: no lesion; 1: mild lesions consisted of multifocal areas of inflammation; 2: moderated lesions consisted of areas of inflammation and necrosis. For each animal, a numerical value was obtained by summing all the scores.

### 2.8 Statistical analysis and graphic creation

Statistical analyses were performed in IBM SPSS Statistics for Windows, version 287.0. 1. 1 (15; IBM Corp., Armonk, N.Y., USA). Significant differences in all tests were considered when *p*-value was <0.05.

For comparisons across the four experimental groups, a non-parametric approach was applied. Kruskal–Wallis tests were used to evaluate overall differences between groups for each variable. When the Kruskal–Wallis test yielded statistically significant results (*p* < 0.05), *post hoc* pairwise comparisons were conducted using the Mann–Whitney *U* test, applying Bonferroni correction where appropriate. If the Kruskal–Wallis test did not reach significance, no pairwise comparisons were performed.

## 3 Results

### 3.1 Immunomodulation of dpB on *S*. Choleraesuis vaccine response

All animals in the control group were seronegative prior to the challenge according to microtiter plate agglutination results. In contrast, animals from all three vaccinated groups presented a detectable serological response, except for one animal in each group. The group that received the *S*. Choleraesuis vaccine alone (group 1) showed the highest antibody titres (median ± standard deviation) [4.38 ± 1.51 ln (e) antibody titres against *S*. Choleraesuis]. Statistically significant differences were observed between each vaccinated group and control group (group 4): inactivated dpB-*Samonella* vaccinated group (group 3) and the control group (Mann–Whitney test using Bonferroni *post hoc* adjust M–W_groups1–4_, *p* = 0.002, M–W_groups 2–4_ *p* = 0.047, M–W_groups 3–4_ *p* = 0.016), whereas no significant differences were found among the three vaccinated groups (Kruskal–Wallis test, *p* > 0.05; Figure 2A).

**Figure 2:**
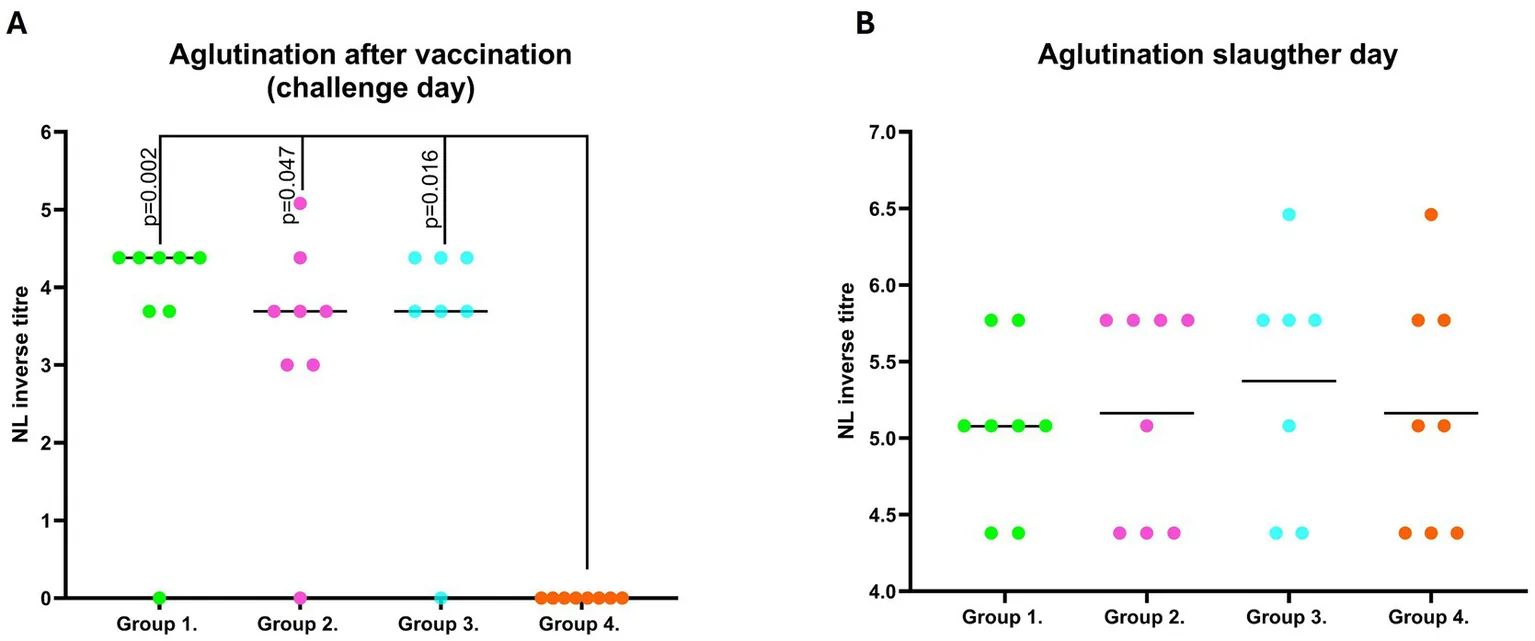
Graphical representation of serological titration of anti-*S*. Choleraesuis antibodies for animals from groups 1 to 4 (G1. *S*. Choleraesuis vaccine, G2. dpB-*S*. Choleraesuis vaccine, G3. Inactivated dpB-*S*. Choleraesuis vaccine, G4. Infection control) from blood samples taken in two different points, **(A)** after vaccination, challenge day and **(B)** in the day of slaughter. Horizontal lines represent median values of each group. NL, Napierian logarithm. *p* values indicated Mann–Whitney test using Bonferroni *post hoc* adjust. Created with GraphPad Prism 10 software.

Vaccinated group (group 1) exhibited the best phagocytic responses against *S*. Choleraesuis (3.23 × 10^4^ ± 8.4 × 10^4^ CFU/mL of blood). In contrast, group 2 (live dpB—*S*. Choleraesuis vaccine) had the lowest phagocytosis activity (1.53 × 10^5^ ± 2.85 × 10^5^ CFU/mL).

For the pre-challenge sera, the stimulation with *S*. Choleraesuis revealed a significant increase in IL-1β levels in the vaccinated-only animals (group 1) compared to live dpB group-*S*. Choleraesuis vaccine (group 2; M–W_Bonferroni *post hoc*_, *p* = 0.04). Additionally, the live dpB group (group 3) exhibited lower concentrations of TNF-α and IL-10 than other groups, while IL-6 levels were notably higher (K–W, *p* > 0.05; Figure 3).

**Figure 3:**
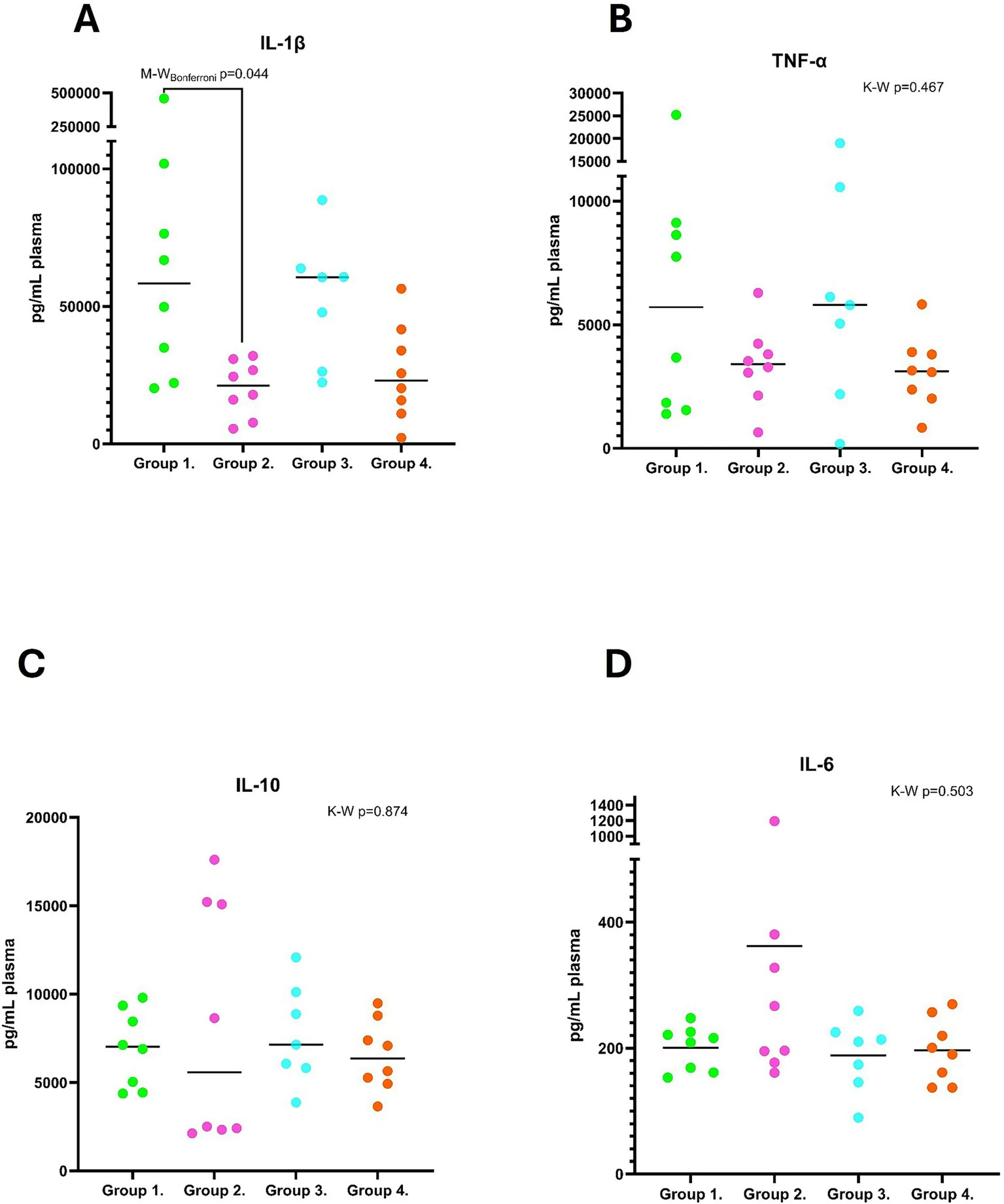
Graphical representation of pre-infection plasmatic cytokines **(A)** IL-1β, **(B)** TNF-α, **(C)**, IL-10 and **(D)** IL-6 results (pg/mL) from blood stimulated with *S*. Choleraesuis from groups 1 to 4 (G1. *S*. Choleraesuis vaccine, G2. dpB-*S*. Choleraesuis vaccine, G3. Inactivated dpB-*S*. Choleraesuis vaccine, G4. Infection control). M–W_Bonferroni_: Mann–Whitney test using Bonferroni *post hoc* adjust. Horizontal lines represent median values of each group. K–W: Kruskall–Wallis test. Created with GraphPad Prism 10 software.

### 3.2 Protection combining dpB and *S*. Choleraesuis inactivated vaccine against *S*. Choleraesuis challenge in swine

Following challenge, all animals in the control group showed detectable antibody titres. Thus, for all groups, antibody titres were significantly higher after the challenge compared to post-vaccination values (Wilcoxon test, *p* ≤ 0.02). However, no statistically significant differences in antibody titres were observed among the four groups (K–W, *p* > 0.05; Figure 2B).

Neither clinical signs nor gross lesions were observed for any of the animals in any of the experimental groups. Bronchitis and interstitial pneumonia were observed in all animals except one animal from group 1. Those lesions were mild to severe in live dpB group 2 and infection control group 4 and consisted of thickening of the lung septa due to the presence of an interstitial inflammatory infiltrate, mainly composed of lymphocytes and macrophages (Figure 4). In contrast, *S*. Choleraesuis vaccine group 1 and inactivated dpB group 3 showed mild to moderate lesions in lungs (Figure 4). Specifically, only three animals from group 1 and one from group 3 showed lesions classified as moderate. Congestion, alveolar oedema and hemorrhage were also consistently observed in all pigs. On the other hand, hepatitis was observed in the liver of all animals but two pigs from group 1 and one from group 3. Moderated lesions in liver with associated necrosis were observed in one animal from *S*. Choleraesuis vaccine group 1 and live dpB group 2, no animal from inactivated dpB group 3 and six pigs from infection control group 4 (Figure 5). The remaining animals showed mild lesions in this organ.

**Figure 4:**
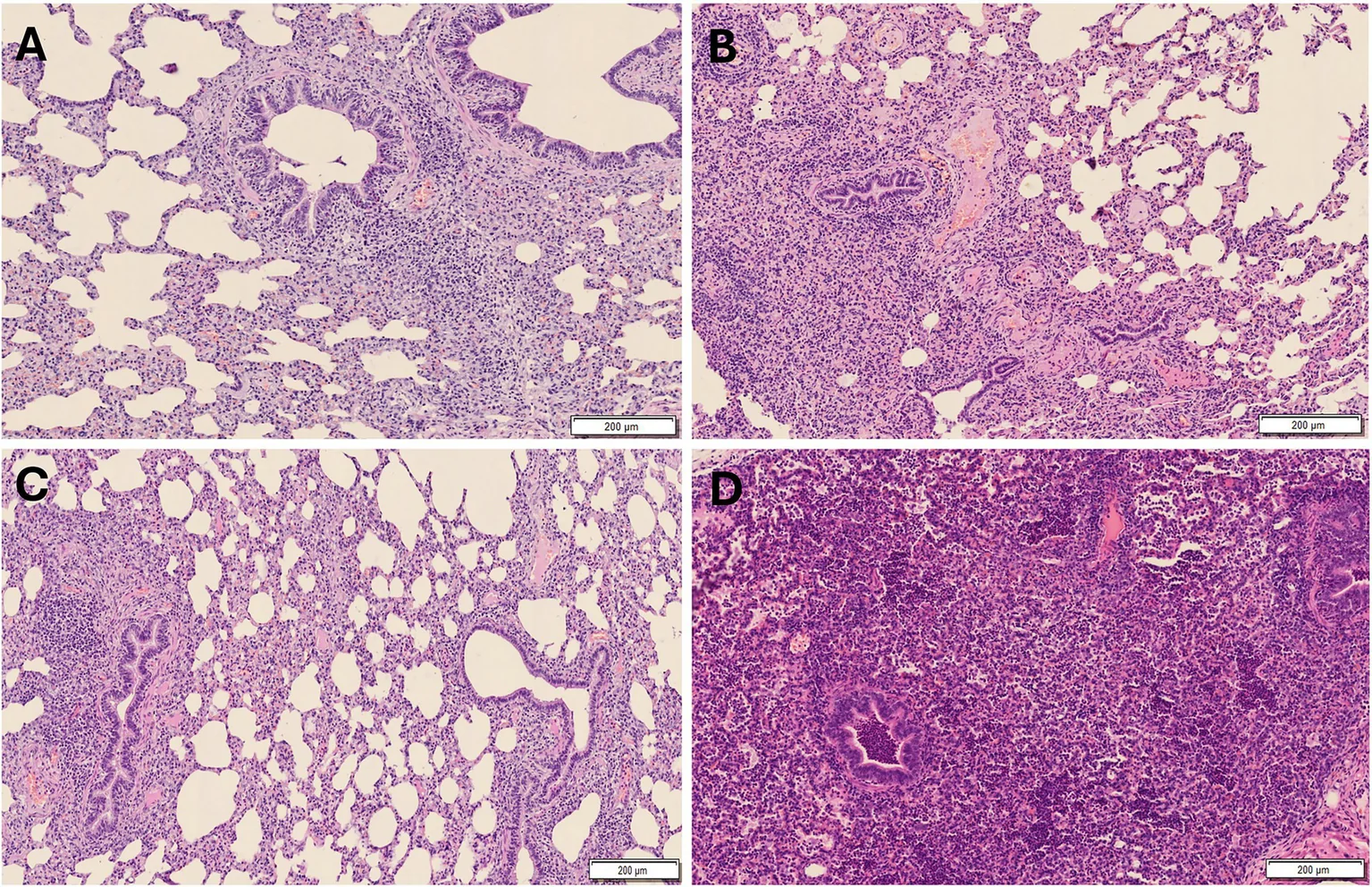
Representative histopathological lesions in lungs of pigs from *S*. Choleraesuis vaccine group 1 **(A)**, live dpB group 2 **(B)**, inactivated dpB group 3 **(C)** and infection control group 4 **(D)**. Lungs present a mild (groups 1 and 3), moderate (group 2) and severe (group 4) bronchiolitis and interstitial pneumonia, composed of mixed interstitial inflammatory infiltrate, mainly consisted of macrophages and lymphocytes, that cause thickening of the septa in different degrees. In image **D**, neutrophils can be also observed. Hematoxylin and eosin stain.

**Figure 5:**
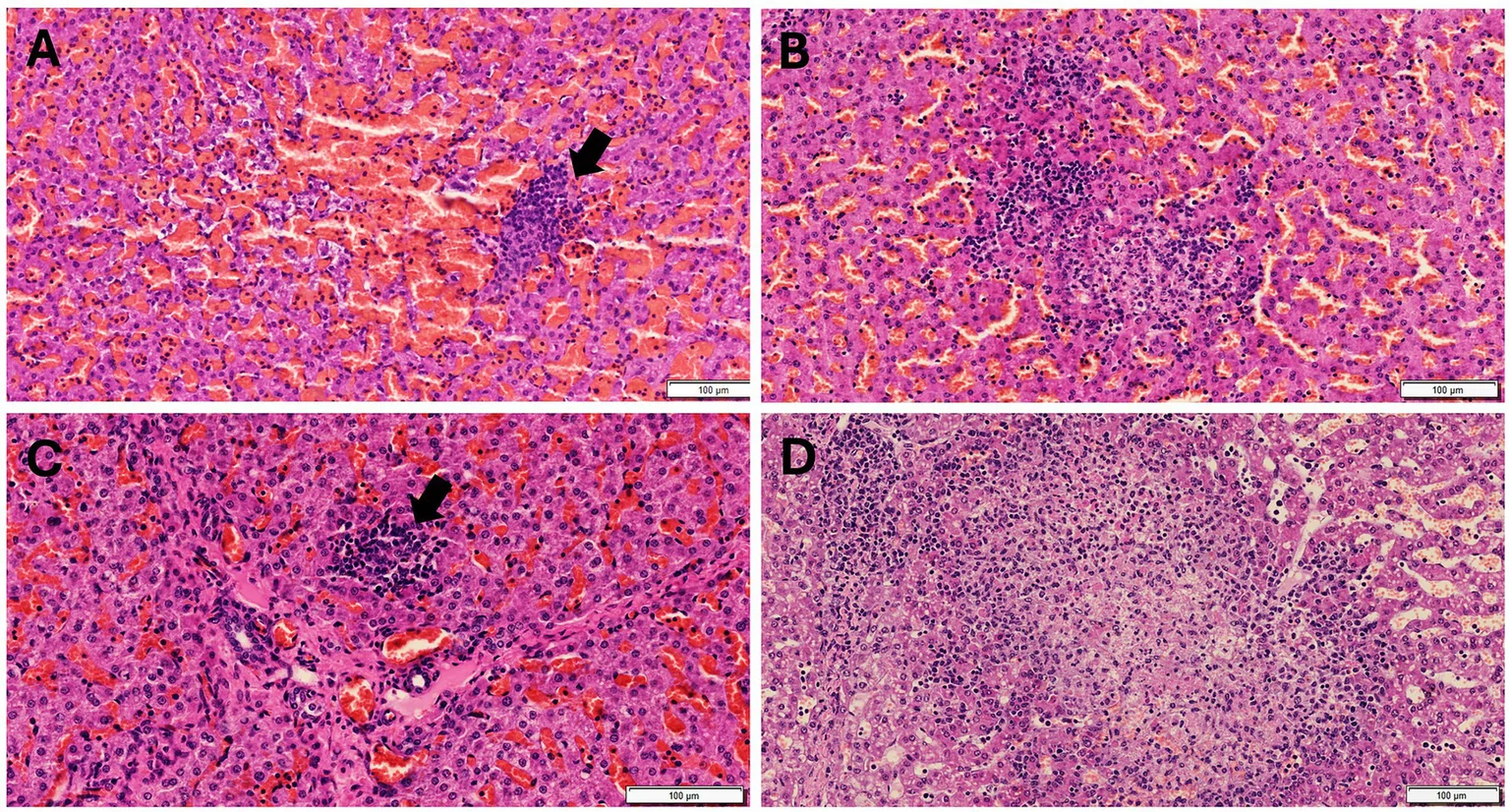
Representative histopathological lesions in liver of pigs from *S*. Choleraesuis vaccine group 1 **(A)**, live dpB group 2 **(B)**, inactivated dpB group 3 **(C)** and infection control group 4 **(D)**. Livers present a mild (groups 1 and 3) and moderate (groups 2 and 4) hepatitis, with an inflammatory infiltrate mainly composed of macrophages and lymphocytes (arrows). In moderate lesions necrosis can be also observed **(B,D)**. Hematoxylin and eosin stain.

A total histopathology score was calculated for each animal by summing the scores from both organs. When analyzing this total score between groups, group 1 (*S*. Choleraesuis vaccine) exhibited significantly lower values when compared to Group 4 (control group; M–W, *p* = 0.017; Figure 6). When evaluating lung histopathological score, group 2 (live dpB—*S*. Choleraesuis vaccine) presented the highest values (Figure 6; K–W, *p* > 0.05).

**Figure 6:**
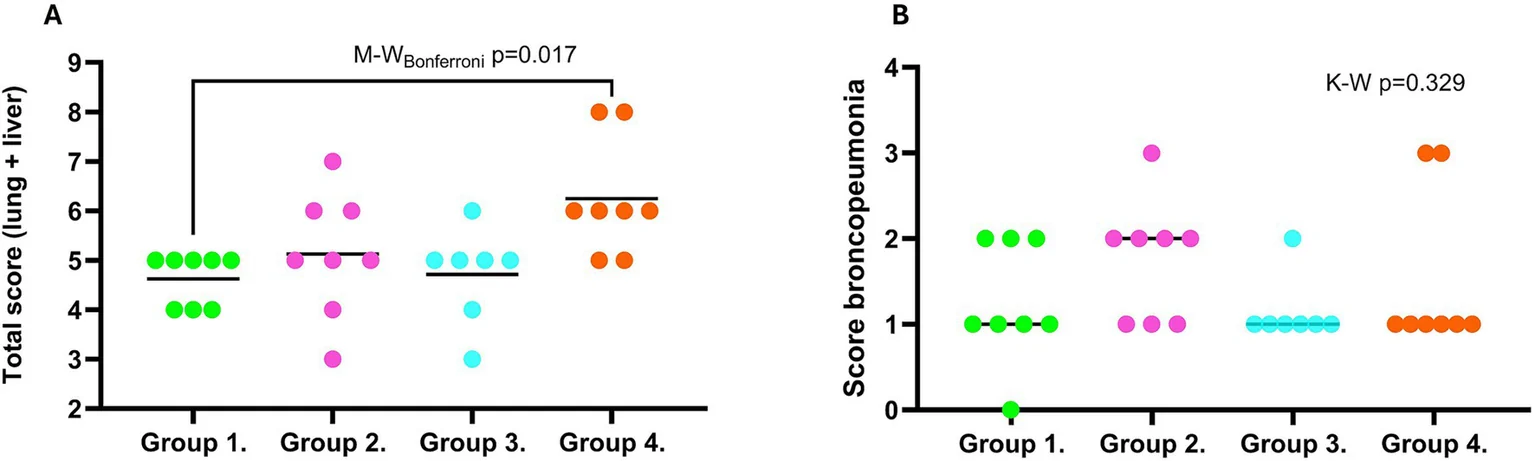
Histopathological evaluation of animals from the experimental groups (G1. *S*. Choleraesuis vaccine, G2. dpB-*S*. Choleraesuis vaccine, G3. Inactivated dpB-*S*. Choleraesuis vaccine, G4. Infection control) including **(A)** total score (composed of lung and liver) and **(B)** bronchopneumonia score in lungs. Created with GraphPad Prism 10 software.

Regarding bacteriological results after infection, for the qualitative analysis, when combining all sampled tissues (cecum content, ileocecal lymph node, lung, and tonsil), groups 1 (*S*. Choleraesuis vaccinated group) and 4 (infected control) presented the highest number of positive samples (50%/51.6%), while groups combining dpB and vaccination (live dpB and inactivated dpB) showed similar lower proportions (34.4%/35.7%; Table 2). No statistically significant differences were detected between groups (K–W, *p* > 0.05). For all groups, the samples with the highest percentage of isolation were ileocaecal lymph node, followed by cecum content.

**Table 2:** Qualitative detection of *S*. Choleraesuis across sampled tissues (cecum content, ileocecal lymph node, lung, and tonsil) in the different experimental groups (G1. *S. Choleraesuis* vaccine, G2. dpB—*S*. Choleraesuis vaccine, G3. Inactivated dpB—*S*. Choleraesuis vaccine, G4. Infection control).

Experimental groups	Ileocecal lymph node +/total	Cecum content +/total	Lungs +/total	Tonsils +/total	Total
G1. *S*. Choleraesuis vaccine	8/8 (1,200 ± 3,610.5 CFU/g)	6/8 (72 ± 3,439.8 CFU/g)	1/8 (0 ± 989.9 CFU/g)	1/8 (0 ± 0 CFU/g)	16/32
G2. Live dpB—*S*. Choleraesuis vaccine	7/8 (1,200 ± 2,481.1 CFU/g)	4/8 (6.5 ± 981.4 CFU/g)	0/8 (0 ± 0 CFU/g)	0/8 (0 ± 0 CFU/g)	11/32
G3. Inactivated dpB—*S*. Choleraesuis vaccine	7/7 (240 ± 2,795.9 CFU/g)	3/7 (0 ± 334.4 CFU/g)	0/7 (0 ± 0 CFU/g)	0/7 (0 ± 0 CFU/g)	10/28
G4. Infection control	7/7 (1,500 ± 878.8 CFU/g)	7/8 (195 ± 3,360.4 CFU/g)	1/8 (0 ± 60.1 CFU/g)	1/8 (0 ± 2,651.7 CFU/g)	16/31

The table shows the proportion of positive samples per group as well as the media ± standard deviation for CFU/g for each sample and group.

Regarding to bacteriological quantitative results (CFU counts) for ileocecal lymph nodes, group 3 (inactivated dpB-*S*. Choleraesuis vaccine) presented the lowest levels. Similarly for cecum content culture counts the lowest levels of CFU were obtained for both groups combining dpB and *S*. Choleraesuis vaccine (group 2 and 3), being the one with the highest values the *S*. Choleraesuis vaccinated group (group 1; Figure 7; Table 2). However, statistically significant differences among groups were not detected (K–W, *p* > 0.05). Regarding the lung and the tonsils, only two and one positive samples were obtained, respectively.

**Figure 7:**
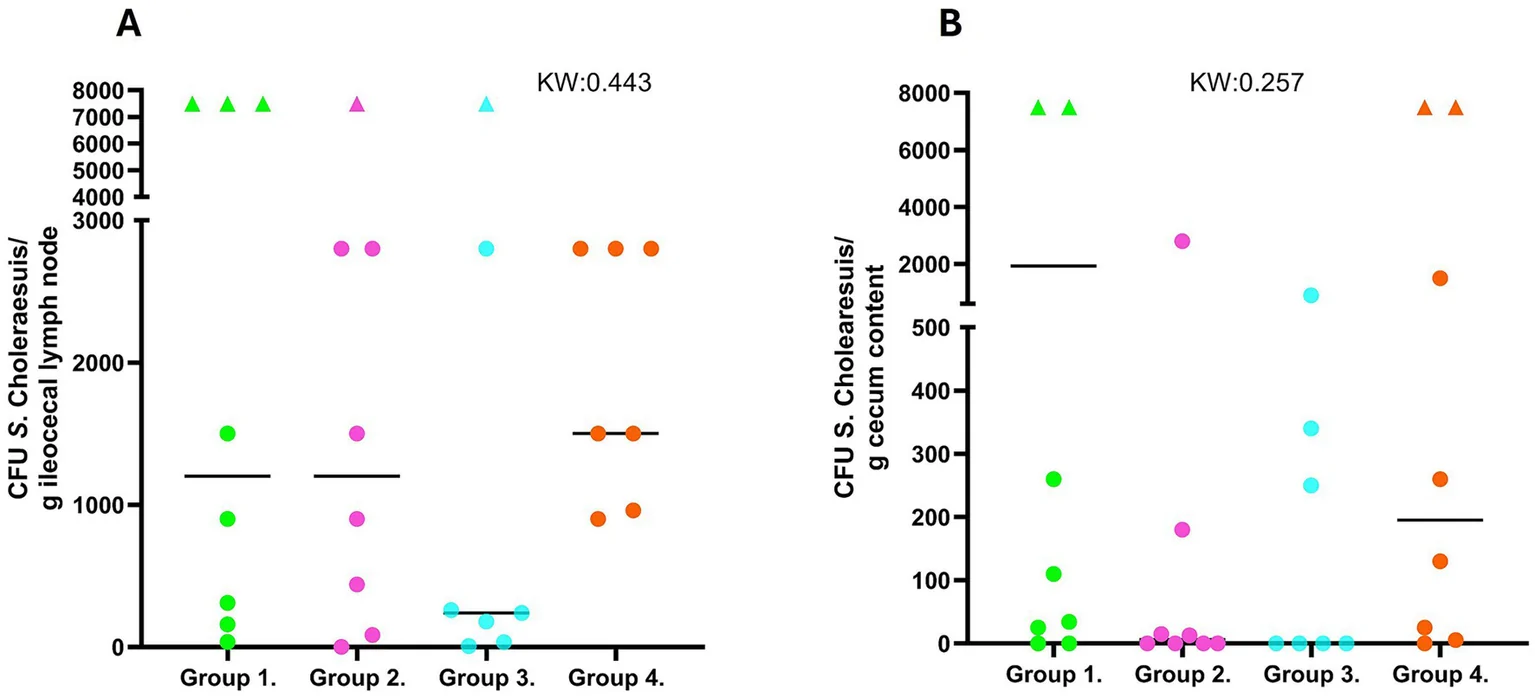
Graphical representation of *S*. Choleraesuis isolation (CFU/g) for animals from groups 1 to 4 (G1. *S*. Choleraesuis vaccine, G2. dpB-*S*. Choleraesuis vaccine, G3. Inactivated dpB-*S*. Choleraesuis vaccine, G4. Infection control) for ileocecal lymph nodes and cecum content. Horizontal black lines represent median values of each group. Triangles represent those samples in which the value could not be estimated because all dilutions evaluated showed growth, so the most conservative *S*. Choleraesuis concentration expected was assigned. K–W: Kruskall–Wallis test. Created with GraphPad Prism 10 software.

### 3.3 Cross-protective immunity against heterologous serotypes: *S*. Typhimurium and its monophasic variant

Prior to challenge, the *S*. Choleraesuis vaccinated group (group 1) exhibited the highest phagocytic responses for *S*. Typhimurium (Figure 8A) and its monophasic variant (Figure 8B). In contrast, group 2 (live dpB—*S*. Choleraesuis vaccine) had the lowest phagocytosis activity for both serotypes (7.28 × 10^5^ ± 4.20 × 10^6^ vs. 1.70 × 10^6^ ± 4.28 × 10^6^ CFU/mL). Statistically significant differences were observed only between *S*. Choleraesuis vaccinated group (group 1) and live dpB-*S*. Choleraesuis vaccine when stimulated with *S*. Typhimurium (M–W, *p* = 0.02). Phagocytosis results were consistent for the three tested *Salmonella* serotypes.

**Figure 8:**
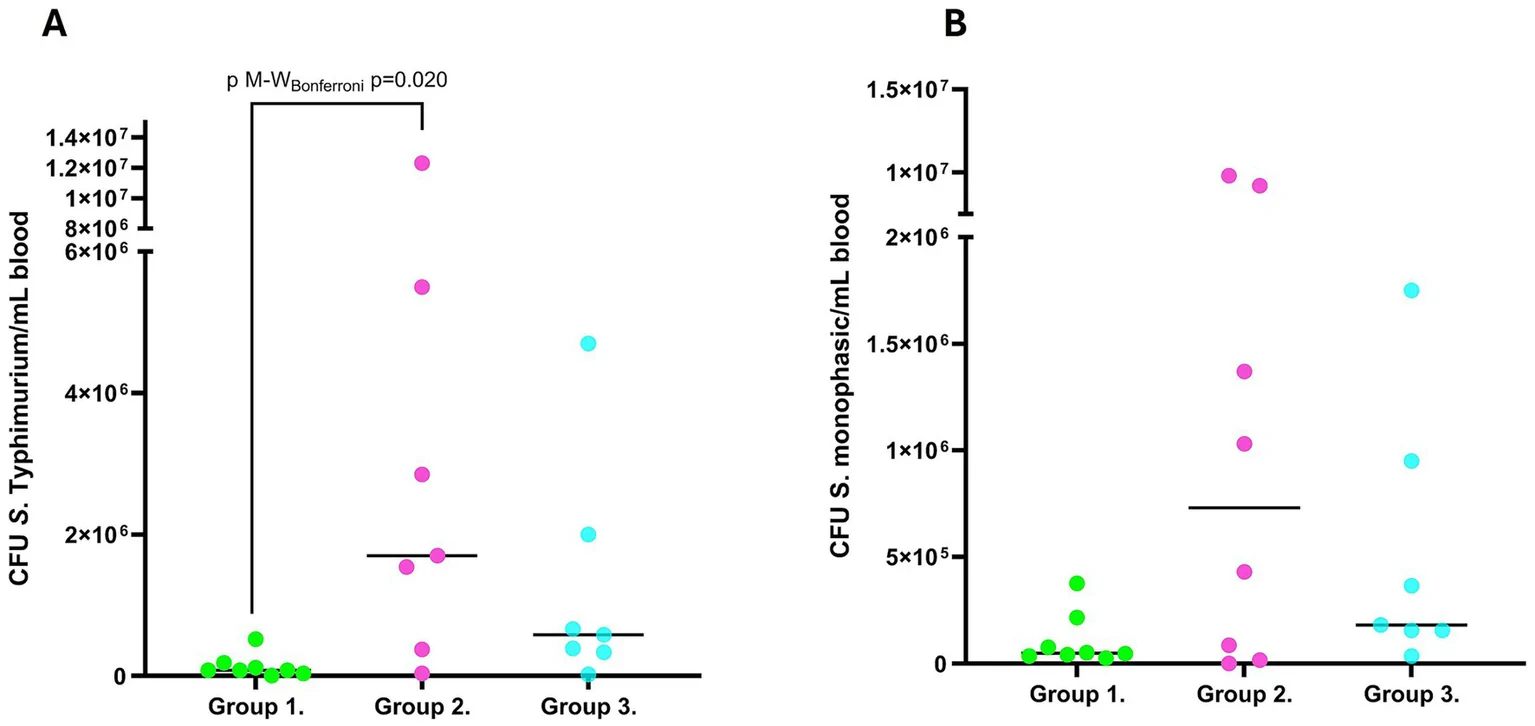
Graphical representation of the results for phagocytosis for **(A)** *S*. Typhimurium and **(B)** its monophasic variant (mL/CFU of blood) performed at day 55 (before challenge) from groups 1 to 3 (G1. *S*. Choleraesuis vaccine, G2. dpB-*S*. Choleraesuis vaccine, G3. Inactivated dpB-*S*. Choleraesuis vaccine, G4. Infection control). Horizontal black lines represent median values of each group. Created with GraphPad Prism 10 software.

Following stimulation with the monophasic variant, IL-10 production was significantly lower in the live dpB-*Salmonella* vaccine group (group 2) compared to the *S*. Choleraesuis vaccinated group (group 1; M–W_Bonferroni *post hoc*_, *p* = 0.03). As with *S*. Choleraesuis, IL-6 levels remained elevated in the group 2 relative to other groups, although no significant differences were found for IL-1β or TNF-α.

In response to *S*. Typhimurium, similar cytokine pattern was observed but did not reach statistical significance. The control group (group 4) consistently showed the weakest cytokine responses (IL-1β, IL-6, TNF-α), followed by the live dpB-*S*. Choleraesuis vaccine group (group 2). IL-10 levels tended to be lower in both dpB-immunized groups than in controls, though differences were not statistically significant (K–W, *p* > 0.05).

When comparing responses within each group (Figure 9), all the three *Salmonella* strains evaluated induced higher IL-6 production compared to PBS, regardless of immunization status. Notably, *S*. Choleraesuis consistently induced the highest IL-6 levels in all groups, with significantly greater responses than both the PBS (Wilcoxon test using Bonferroni *post hoc* adjust, *p* < 0.001) and the monophasic variant in the dpB-immunized animals, both live dpB (Wilcoxon, *p*_Bonferroni_ = 0.012) and inactivated dpB (Wilcoxon, *p*_Bonferroni_ = 0.043). *S*. Typhimurium also triggered significantly higher IL-6 responses than PBS in the *S*. Choleraesuis vaccine group (group 1; Wilcoxon, *p*_Bonferroni_ < 0.022) and inactivated dpB-Salmonella vaccine group (group 3; Wilcoxon, *p*_Bonferroni_ = 0.043).

**Figure 9:**
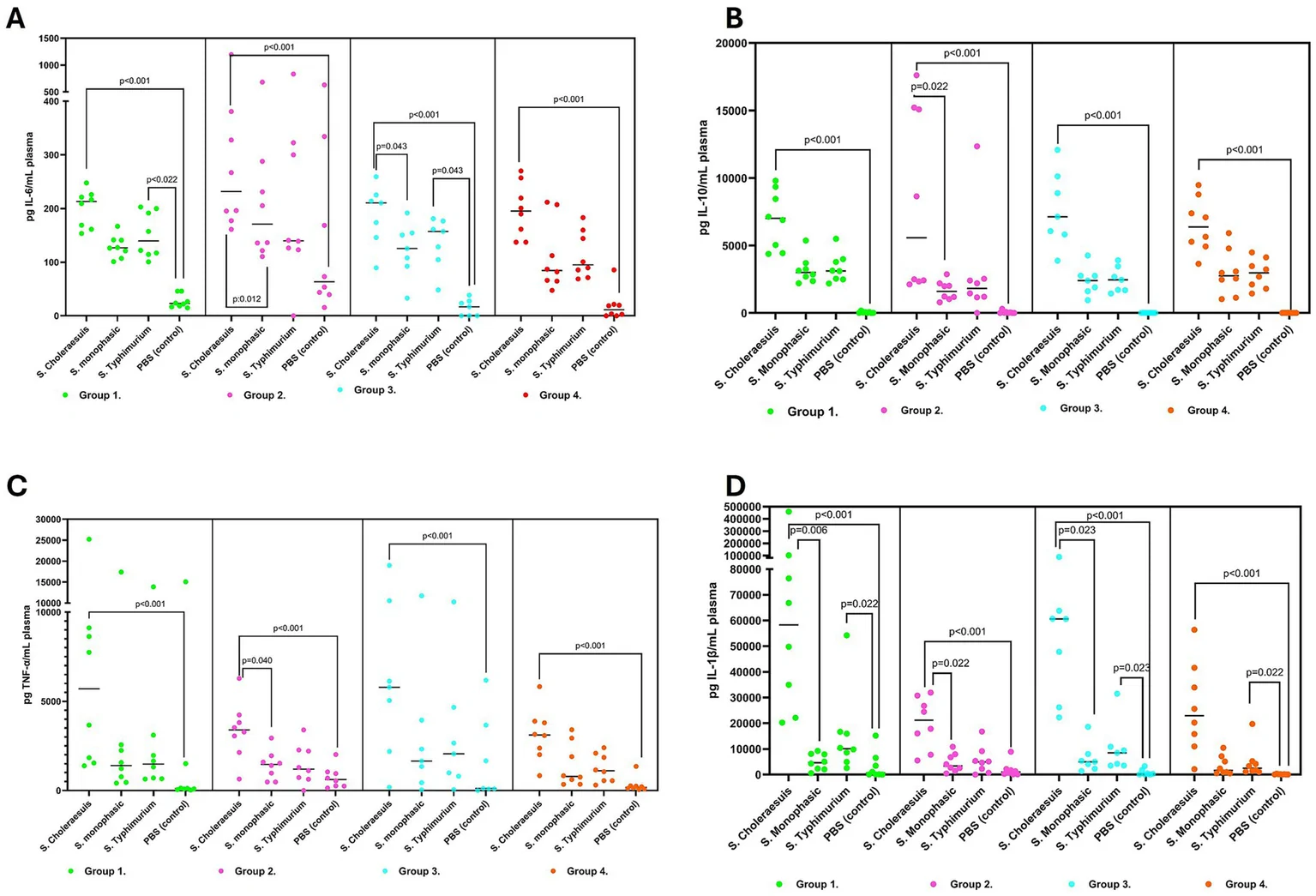
Graphical representation of pre-infection cytokines **(A)** IL-6, **(B)** IL-10, **(C)** TNF-α and **(D)** IL-1β results (pg/mL) in sera stimulated with *S*. Choleraesuis, *S*. Typhimurium, its monophasic variant and PBS used as negative control from animals in groups 1 to 4 (G1. *S*. Choleraesuis vaccine, G2. dpB-*S*. Choleraesuis vaccine, G3. Inactivated dpB-*S*. Choleraesuis vaccine, G4. Infection control). Created with GraphPad Prism 10 software.

A similar intra-group pattern was observed for TNF-α, IL-10, and IL-1β, with *S*. Choleraesuis (Figures 9B–D) consistently inducing the strongest responses and significantly higher than PBS stimulation in all groups (Wilcoxon test, *p*_b_ < 0.001). For IL-1β, responses to the monophasic variant were particularly low and often resembled the PBS condition. However, for *S*. Choleraesuis all three groups [*S*. Choleraesuis vaccine (group 1; Wilcoxon, *p*_B_ = 0.006), live dpB-*S*. Choleraesuis vaccine (group 2; Wilcoxon, *p*_B_ = 0.022), inactivated dpB-*S*. Choleraesuis vaccine (group 3; Wilcoxon, *p*_B_ = 0.023)] induced significantly higher IL-1β levels than the monophasic variant.

After challenge, no differences were found among the four groups for any of the cytokines tested (K–W, *p* > 0.05). IL-6 levels tended to be highest in group 2 and lowest in group 3. A similar trend was observed for IL-10, although most values were below detection limits.

## 4 Discussion

In this study, we evaluated, for first time, the immunological, bacteriological and pathological outcomes of combined strategies based on trained immunity (live or inactivated dpB) together with a conventional vaccine against *S*. Choleraesuis followed by a *S*. Choleraesuis challenge. Our objective was to evaluate both the combination of innate and acquired immunity against *S*. Choleraesuis infection and the potential cross-protection against heterologous serotypes, specifically *S*. Typhimurium and the monophasic variant.

As expected, the conventional vaccine against *S*. Choleraesuis alone (group 1) induced a robust humoral response prior to exposure [consistent with previous data on the immunogenicity of inactivated *S*. Choleraesuis vaccines (25, 26)], with slightly higher antibody titres than the groups that received dpB in combination with the vaccine. In line with this, group 1 also showed a significant reduction in total histopathological score compared to the unvaccinated infected control group. However, this group showed the highest bacterial loads in cecum content and tissues, highlighting that strong humoral responses do not necessarily correlate with effective bacterial clearance. This reflects the classical dissociation between antibody immunogenicity and functional protection that has been reported in *Salmonella* and other bacterial infections (27).

Interestingly, the group that received inactivated dpB together with the vaccine (group 3) showed a trend toward lower bacterial recovery, with the lowest CFU counts in both feces and ileocecal lymph nodes and higher antibody titers at the time of sacrifice, as well as less severe lesions in lung and liver. These results suggest that the inclusion of inactivated dpB in immunization protocols, may contribute to local immune responses or modulate innate immunity in a way that could favor bacterial control. Indeed, this group maintained a balanced cytokine profile including both IL-1β and TNF-*α*, with no excessive damage as observed in group 2 (28). This balanced activation may be compatible with the concept of “trained tolerance,” in which the innate immune system is epigenetically reprogrammed not only to respond stronger but also to regulate inflammation more efficiently, thereby avoiding collateral tissue damage. This result could indicate a more favorable interaction between vaccination and the inactivated immunomodulator than with the live formulation, since inactivated have been reported to induce trained immunity through both epigenetic and metabolic rewriting (29, 30). An opposite pattern was reported by Vaz-Rodrigues et al. (19) in which oral administration of HIMB significantly reduced pulmonary lesions after *S*. Choleraesuis challenge but failed to decrease tissue colonization. This divergence suggests that the different inactivated mycobacterial immunomodulators can favor different effector pathways. These discrepancies might also be influenced by factors such as the specific mycobacterial strain used, the route and timing of administration, or even the dosage applied which are variables known to affect the induction of trained immunity, and should be considered when comparing outcomes across different studies (31).

In contrast, the group that combined live dpB with *S*. Choleraesuis vaccine (group 2) showed the poorest phagocytic activity and the most pronounced lung lesions as well as a proinflammatory cytokine profile defined by elevated IL-6 but reduced IL-10 and TNF-*α* just before challenge. This could reflect a dysregulated immune response, which converges in inflammation and higher tissue damage (32). Specifically, the high IL-6 values have been associated with bacterial infections due to its proinflammatory amplification (33). It is also possible that overstimulation of innate responses by the live immunomodulator induced a state similar to endotoxin tolerance or functional exhaustion of myeloid cells, which would contribute to the observed reduction in phagocytic capacity (34). The deficient phagocytic response of group 2 is particularly relevant when considering the role of opsonisation in effective phagocytosis (35). Opsonins, such as specific antibodies and complement components, particularly C3b, C1q, and C4b-derived fragments, are essential for marking pathogens and enhancing their recognition by phagocytes through receptors such as CR1 (CD35) and CR3 (CD11b/CD18) (36, 37). In our study, group 1 presented the strongest humoral response before challenge, which may have contributed greater opsonisation, and, consequently, greater phagocytic activity. In contrast, the reduction in antibody titres in group 2 may have limited the availability of opsonins, resulting in poor recognition of pathogens. This connection between humoral immunity and effective phagocytosis has been well documented in other bacterial infections, where deficient opsonisation correlates with poor pathogen clearance (38). These findings underline the importance of considering the interaction between innate and adaptative immunity when evaluating protective responses.

Phagocytosis and cytokine assays using heterologous serotypes (*S*. Typhimurium and the monophasic variant), although weaker, revealed consistent trends with those observed in *S*. Choleraesuis. This may indicate the presence of cross-reactive immune responses, although evidence of cross-protection was not directly assessed *in vivo*. Such cross-reactivity is most likely mediated by conserved pathogen-associated molecular patterns (PAMPs), such as LPS or flagellin, which activate pattern-recognition receptors (TLR4, TLR5, NOD-like receptors) and provide a functional memory independent of adaptive specificity (39–41).

Previous studies have shown that heat-inactivated *Mycobacterium bovis* (HIMB) can induce partial protection against *S*. Choleraesuis in pigs in the absence of vaccination (19), suggesting that mycobacterial preparations can induce trained immunity in swine. This supports the reason for exploring dpB as a potential inducer of innate immune training. Although the present study cannot confirm trained immunity since epigenetic markers were not assessed and a dpB only group was not included, our findings are partially consistent with the immunomodulatory patterns described for mycobacteria in other models. Importantly, several adjuvants, including β-glucan and mycobacterial-derived formulations, have shown to induce trained immunity (42). Therefore, a possible capacity of dpB to modulate innate responses compatible with trained immunity should be considered in this study.

This study has several limitations. First, the relatively small number of animals per group (*n* = 8, 7 in group 3), which was a decision based on the 3Rs principle (replacement, reduction, and refinement), aiming to minimize animal use while still obtaining meaningful results. Also, the evaluation of cross-reactive immunity to heterologous *Salmonella* serovars was done using *ex vivo* assays. Although an *in vivo* challenge would have provided stronger evidence of cross-protection, we considered this approach a valuable first step to explore the potential of trained immunity, especially within the ethical limits of reduction. Another limitation is that groups treated only with inactivated or live dpB were not included, which would have helped to evaluate their possible individual effects. However, previous results already gave us useful information about their behavior on their own, which allowed us to focus on the most relevant combinations while avoiding additional excessive animal use (43).

In conclusion, our results suggest, for first time, that combining inactivated dpB and a conventional *S*. Choleraesuis vaccine may offer some exploratory indications of advantage, potentially contributing to reduced bacterial shedding and tissue colonization. However, it is important to note that most of the observed differences were modest and did not reach statistical significance. On the other hand, the use of live dpB seemed to interfere with some protective responses, possibly due to an overly strong or unbalanced innate activation (31, 44, 45). To the best of our knowledge, this is the first time that a porcine-derived mycobacterial immunomodulator has been tested in combination with a commercial vaccine in an exploratory attempt to assess wheter compatible mechanisms with trained immunity could be induced in swine. While our findings are preliminary and require further confirmation, they suggest that combining immunomodulatory strategies with conventional vaccination programs. Further research and larger trials are needed to clearly establish the relevance and reproducibility of these effects under field conditions.

## Data Availability

The original contributions presented in the study are included in the article/supplementary material, further inquiries can be directed to the corresponding author.
